# Supplementary material for: TAD fusion score: discovery and ranking the contribution of deletions to genome structure
Source: Genome Biol. 2019 Mar 21;20:60. doi: 10.1186/s13059-019-1666-7 (PMC6427865; doi:10.1186/s13059-019-1666-7)
Supplement: Supplementary file 1 — Supplementary figures and tables. (PDF 10,614 kb) [file 13059_2019_1666_MOESM1_ESM.pdf]

Supplementary Figures and Tables for "TAD-fusion score: discovery and ranking the contribution of deletions to genome structure"

Linh Huynh[1] and Fereydoun Hormozdiari[1,2,3,*]

[1] Genome Center, UC Davis [2] UC Davis MIND institute
[3] Department of Biochemistry and Molecular Medicine, UC Davis

[*]contact: fhormozd@ucdavis.edu

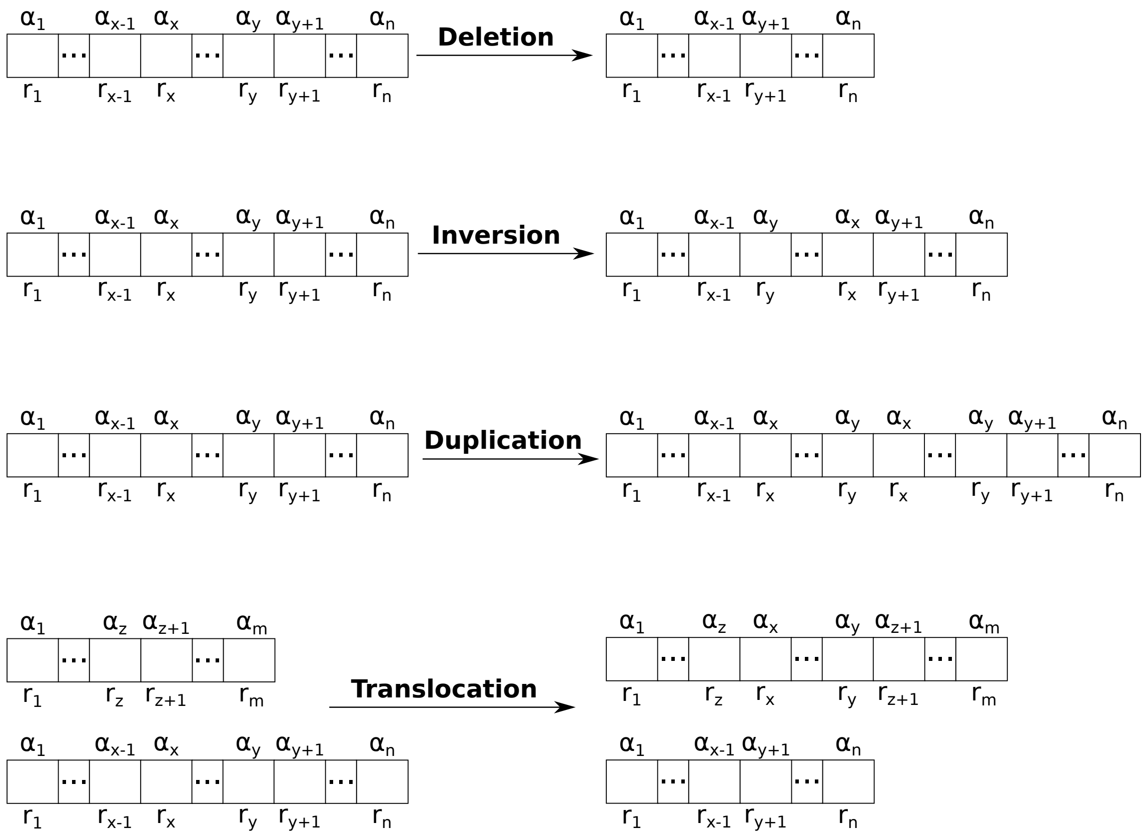

Figure S1: An illustration on how we can predict the genome structure change due to SVs by our model. In our model, the interaction between any two bins i and j only depends on: (i) The bias of these bins (i.e. α_i, α_j) (ii) genomic distance (i.e. how many bin between them), (iii) The insulator/separator of bins between them (i.e. the sum of all r_k of bins k between i and j). We assume that all parameter values (α, β, r) will not be changed due to SVs. Thus only factors (ii) and (iii) can be changed by SVs but they can be determined for any SV as in the the figure.

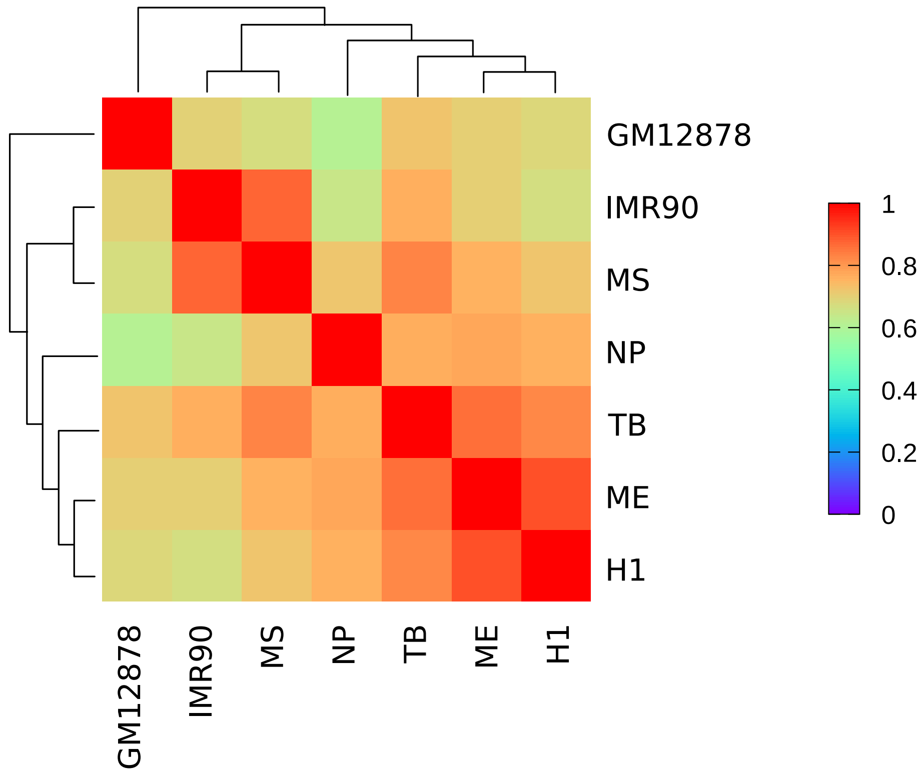

Figure S2: Similarity matrix and hierarchical cluster of the seven cell lines built using the insulation per bin (parameter r_i). This hierarchical clustering is identical to the one reported by [1] using the same cell lines, and similar to the one produced using A/B compartments as reported in [2] (with one minor exception of swap in the order of TB and NP).

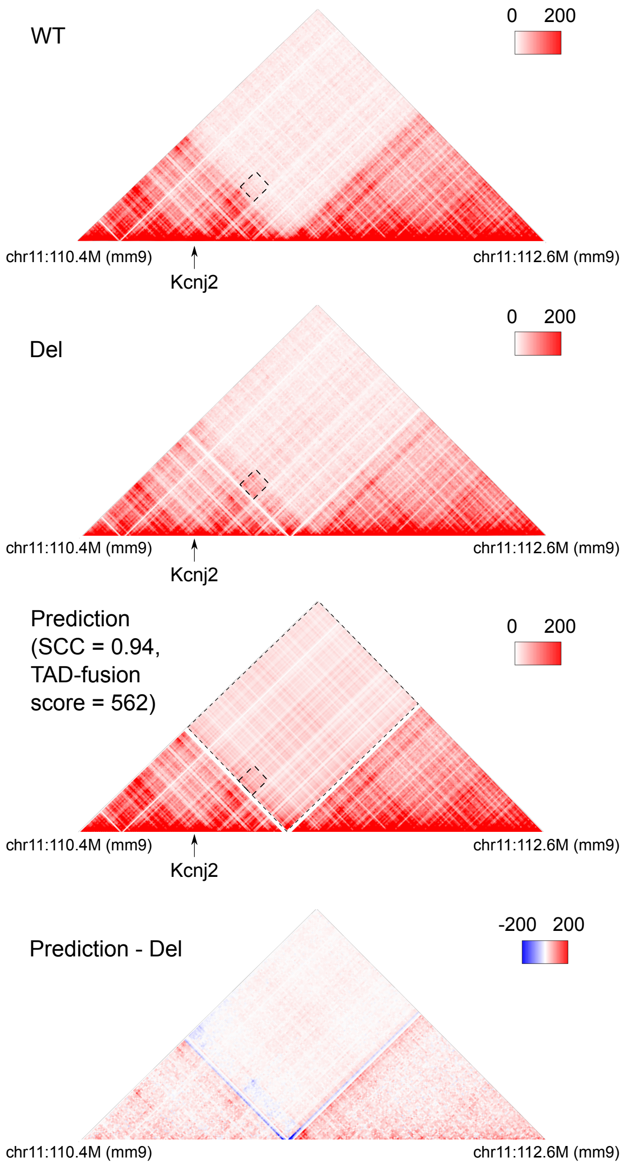

Figure S3: An illustration on the prediction of Hi-C change due to the deletion at Kcnj2-Sox9 boundary [3]. The first subplot shows the Hi-C data without the deletion. In the last three subplots, blank lines represent the deletion. We suppose the interaction in the upstream region and the downstream region of the deletion are not changed due to this deletion. Thus we only predict the interaction between a locus in the upstream region and a locus in the downstream region (the rectangle region limited by the dashed line in the third subplot), other interactions are copied from the original data (i.e. from the first subplot). The stratum adjusted correlation coefficient (SCC) is calculated from all values (i.e. the whole triangle) between the experimental Hi-C (the second subplot) and the prediction Hi-C (the third subplot). The TAD-fusion score is calculated for the region limited by the dashed line in the third subplot. The last subplot shows the difference between the prediction (the third subplot) and the experimental data (the second subplot). The small region marked by dashed lines highlights the interaction change between Kcnj2 and loci in the downstream region.

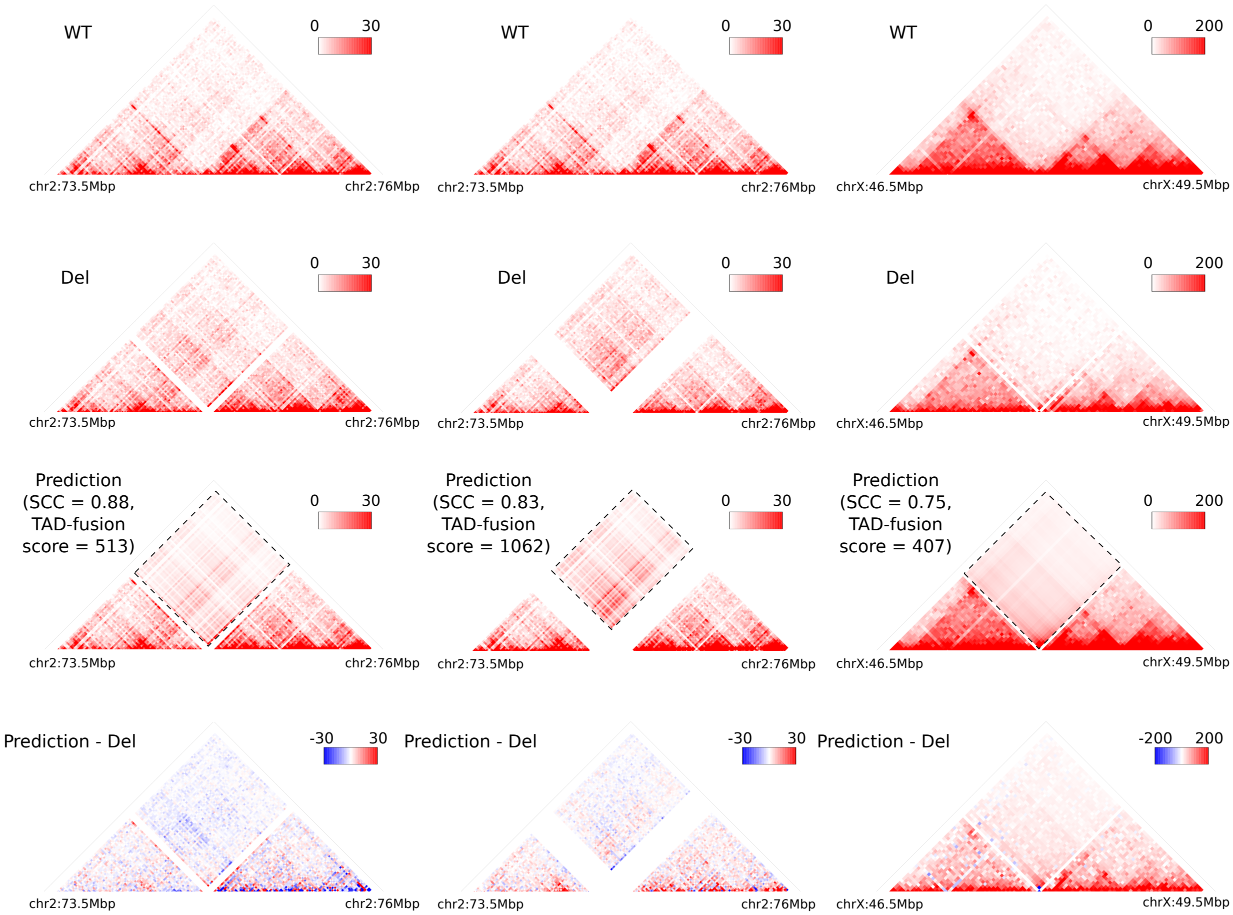

Figure S4: An illustration on the prediction of Hi-C change due to the deletion at HoxD (the left column and the middle column, [4]) and Firre (the right column, [5]). Annotation is similar to the ones in figure S3.

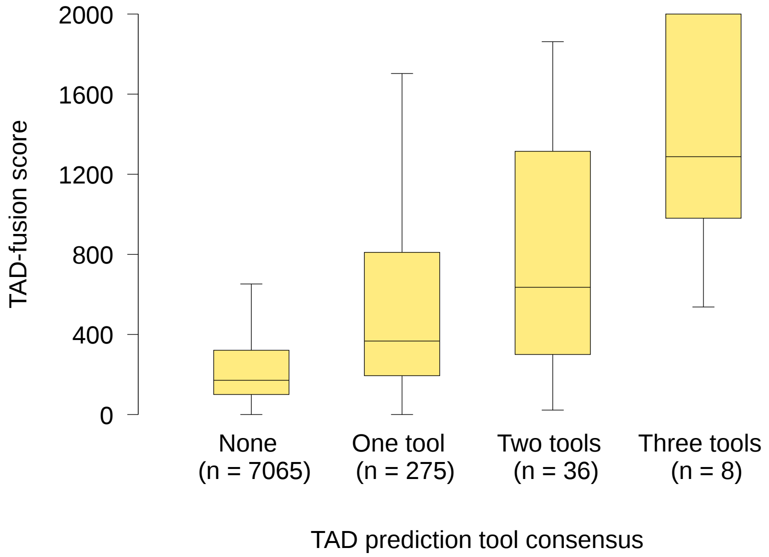

Figure S5: Our TAD fusion score and the prediction consensus (between different TAD callers) of 1KG deletions. Each deletion was classified into one of four groups depending on how many tools predicted that this deletion removed a TAD boundary.

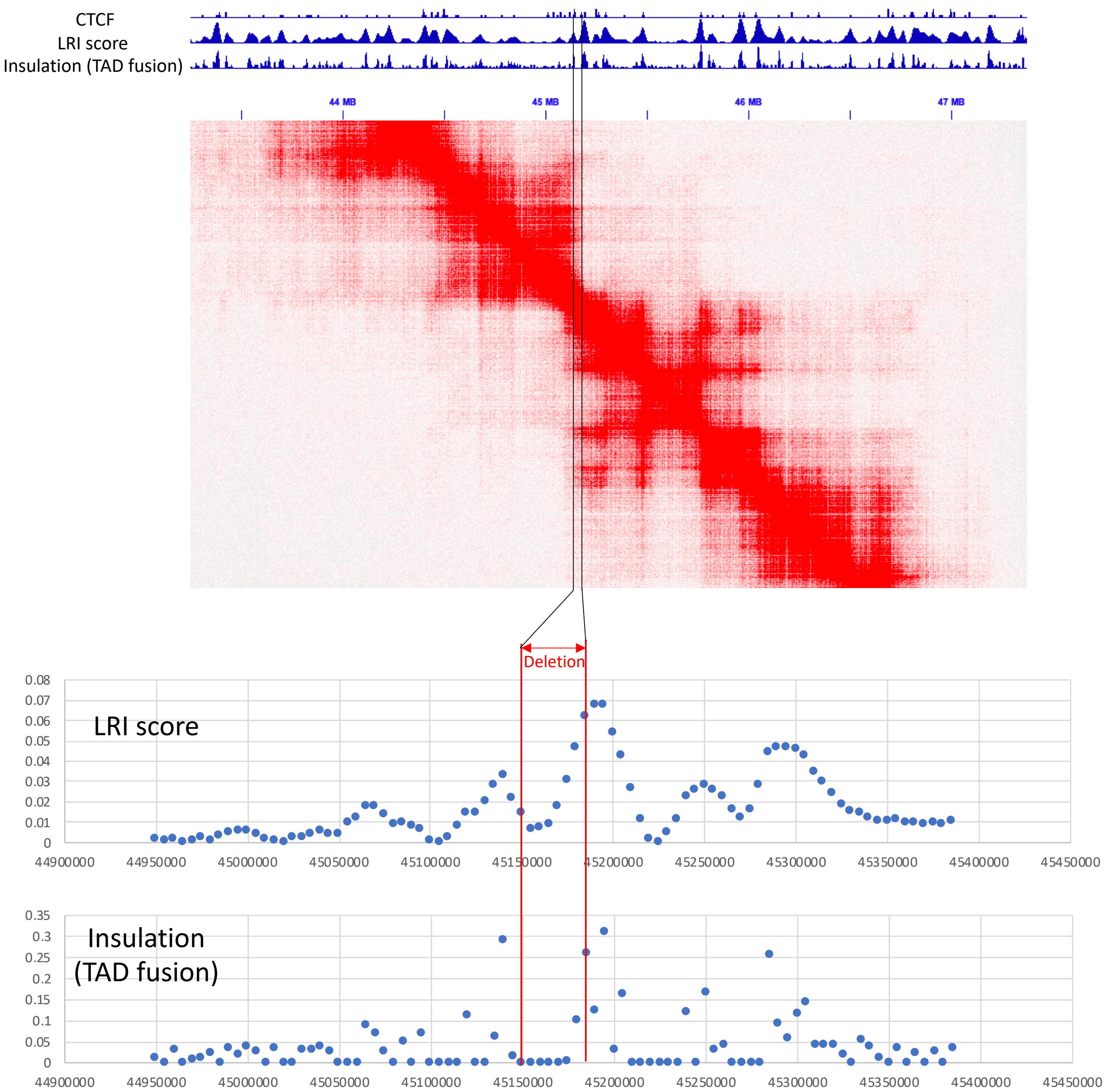

Figure S6: An illustrated example (one of 1KG deletions at chromosome 1) shows the limitation of the scoring method that only uses the maximum insulation score of bins inside the deletion. In that case, this method gives a high score since the deletion removes a bin with a large insulation score (measured by LRI score or our insulation). Our method gives a low score for this deletion since there are still other insulators remaining (both the upstream and downstream of the deletion). The upper image is visualized with Juicebox [6], the lower image visualizes the LRI score and our insulation that are zoomed in around the deletion.

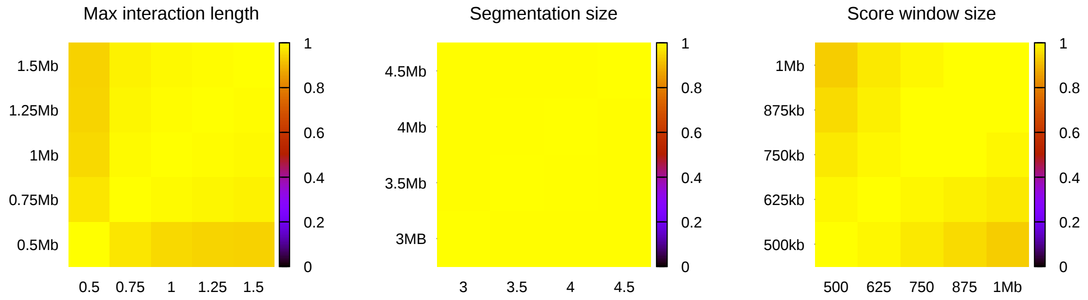

Figure S7: Evaluation of the robustness of our method when the parameter values are changed: Maximum interaction length (left), segmentation length (middle) and the window size of scoring. The robustness is evaluated as the Pearson correlation coefficient between two vectors of the TAD-fusion scores of all 1KG deletions of chromosome 21 calculated with two different parameter values respectively.

Disease	Author	PMID	Chr	Start	End
Limb malformation	Lupianez et al.	25959774	2	221278232	223014332
Demyelinating leukodystrophy	Giorgio et al.	25701871	5	125385998	126046500
Intellectual disability	Redin C et al.	27841880	5	87750000	88150000
Intellectual disability	Redin C et al.	27841880	5	87850000	90250000
Mesomelic dysplasia	Flottmann et al.	26032025	6	19964281	22008341
Mesomelic dysplasia	Flottmann et al.	26032025	6	19974194	22013061
Mesomelic dysplasia	Flottmann et al.	26032025	6	19849280	21694600
Mesomelic dysplasia	Flottmann et al.	26032025	6	19153386	21698497

Table S1: Deletions curated from the literature that can cause the disease by changing the 3D chromosome structure.

Tools	Setting
Arrowhead	TAD list is the file GSE63525_GM12878_primary+replicate_Arrowhead_domainlist.txt.gz from GEO data GSE63525.
CaTCH	The input is the normalized Hi-C data (resolution = 5kb, normalized by SQRTVCnorm), we set RI threshold = 0.65 as the author suggested.
InsulationScore	The input is the normalized Hi-C data (resolution = 5kb, normalized by SQRTVCnorm). We tried different value combinations (250kbp, 50kbp), (250kbp, 100kbp), (500kbp, 100kbp) and (500kbp, 200kbp) for the insulation square and the insulation delta respectively. A bin is a TAD boundary if the insulation score >= 0.7. The result in the main text is of the case (500kbp, 200kbp).
CDB (LRI)	There is no parameter, we only follow the manual.

Table S2: Parameter setting for TAD callers and insulation score tool in the benchmark.

Resource	Source	Identifier
Software		
Genome browser		https://genome.ucsc.edu
ILOG CPLEX		http://www.ilog.com/products/cplex
Insulation-score	[7]	https://github.com/dekkerlab/crane-nature-2015
CaTCH	[8]	https://github.com/zhanyinx/CaTCH_R
Arrowhead	[9]	GSE63525
CDB (LRI)	[10]	https://github.com/ChenFengling/RHiCDB
HiCRep	[11]	https://github.com/MonkeyLB/hicrep
TAD-fusion score	This paper	https://github.com/HormozdiariLab/TAD-fusion-score
Other		
HiC data	[9]	GSE63525
	[1]	GSE87112
HiCCUP loops	[9]	GSE63525
CTCF binding sites	[12]	https://www.encodeproject.org
1KG deletion data	[13]	http://www.internationalgenome.org/data
GreatApe data	[14]	
TCGA deletion data	TCGA	https://portal.gdc.cancer.gov

Table S3: Softwares and datasets used in this study.

		Model						
		H1	ME	NP	TP	MS	IMR90	GM12878
Raw	H1	**0.976**	0.951	0.924	0.894	0.888	0.843	0.707
	ME	0.942	**0.973**	0.901	0.924	0.883	0.865	0.751
	NP	0.936	0.922	**0.972**	0.878	0.882	0.839	0.711
	TP	0.881	0.922	0.85	**0.973**	0.88	0.881	0.813
	MS	0.873	0.882	0.86	0.889	**0.948**	0.892	0.728
	IMR90	0.821	0.853	0.809	0.879	0.879	**0.949**	0.761
	GM12878	0.69	0.743	0.669	0.809	0.713	0.755	**0.972**

Table S4: The SCC score between our estimated Hi-C matrix and raw Hi-C matrix of different cell types.

Disease	Chr	Start	End	Note
Limb malformation (Lupianez et al., PMID:25959774)	2	222800125	223011646	In mice, the deletion DelB (chr1:76388978-78060839, mm9) was validated to cause the limb malformation while the deletion chr1:76388978-77858974 was validated to have no effect on the limb development. Therefore, we inferred that the deletion chr1:77858974-78060839 caused the limb malformation. We lifted this mm9 coordinates to get the the hg19 coordinates chr2:222800125-223011646.
Limb malformation (Lupianez et al., PMID:25959774)	2	220546271	220643226	Similarly, the deletion Dbf (chr1:75098488-75694480, mm9) was validated to cause the limb malformation but the deletion chr1:75098461-75591878 was validated to have no effect on the limb development. Therefore, we inferred that the deletion chr1:75591878-75694480 caused the limb malformation. We lifted this mm9 coordinates to get the the hg19 coordinates chr2:220546271-220643226.
Demyelinating leukodystrophy (Giorgio et al., PMID:25701871)	5	125862217	126010920	The deletion chr5:125385998-126046500 was reported to cause demyelinating leukodystrophy but we found three deletions in 1KG deletion set (chr5:125551305-125566303, chr5:125849235-125862217, and chr5:126010920-126032321) which were supposed to not cause the disease. Therefore, we inferred that the longest sub-deletion chr5:125862217-126010920 that did not overlap with any 1KG deletion caused the disease.
Intellectual disability (Redin C et al., PMID:27841880)	5	87850000	88150000	We inferred this deletion by intersecting two overlapping deletions (chr5:87750000-88150000 and chr5:87850000-90250000) that caused the intellectual disability.

Table S5: Deletions inferred from curated deletions (table S1) that can cause the disease by changing the 3D chromosome structure.

References

[1] A. D. Schmitt, M. Hu, I. Jung, Z. Xu, Y. Qiu, C. L. Tan, Y. Li, S. Lin, Y. Lin, C. L. Barr, *et al.*, "A compendium of chromatin contact maps reveals spatially active regions in the human genome," *Cell reports*, vol. 17, no. 8, pp. 2042–2059, 2016.

[2] J. R. Dixon, I. Jung, S. Selvaraj, Y. Shen, J. E. Antosiewicz-Bourget, A. Y. Lee, Z. Ye, A. Kim, N. Rajagopal, W. Xie, *et al.*, "Chromatin architecture reorganization during stem cell differentiation," *Nature*, vol. 518, no. 7539, p. 331, 2015.

[3] M. Franke, D. M. Ibrahim, G. Andrey, W. Schwarzer, V. Heinrich, R. Schöpflin, K. Kraft, R. Kempfer, I. Jerković, W.-L. Chan, *et al.*, "Formation of new chromatin domains determines pathogenicity of genomic duplications," *Nature*, vol. 538, no. 7624, p. 265, 2016.

[4] E. Rodríguez-Carballo, L. Lopez-Delisle, Y. Zhan, P. J. Fabre, L. Beccari, I. El-Idrissi, T. H. N. Huynh, H. Ozadam, J. Dekker, and D. Duboule, "The hoxd cluster is a dynamic and resilient tad boundary controlling the segregation of antagonistic regulatory landscapes," *Genes & development*, vol. 31, no. 22, pp. 2264–2281, 2017.

[5] A. R. Barutcu, P. G. Maass, J. P. Lewandowski, C. L. Weiner, and J. L. Rinn, "A tad boundary is preserved upon deletion of the ctcf-rich firre locus," *Nature communications*, vol. 9, no. 1, p. 1444, 2018.

[6] N. C. Durand, J. T. Robinson, M. S. Shamim, I. Machol, J. P. Mesirov, E. S. Lander, and E. L. Aiden, "Juicebox provides a visualization system for hi-c contact maps with unlimited zoom," *Cell systems*, vol. 3, no. 1, pp. 99–101, 2016.

[7] E. Crane, Q. Bian, R. P. McCord, B. R. Lajoie, B. S. Wheeler, E. J. Ralston, S. Uzawa, J. Dekker, and B. J. Meyer, "Condensin-driven remodelling of x chromosome topology during dosage compensation," *Nature*, vol. 523, no. 7559, pp. 240–244, 2015.

[8] Y. Zhan, L. Mariani, I. Barozzi, E. G. Schulz, N. Bluthgen, M. Stadler, G. Tiana, and L. Giorgetti, "Reciprocal insulation analysis of Hi-C data shows that TADs represent a functionally but not structurally privileged scale in the hierarchical folding of chromosomes," *Genome Research*, pp. gr–212803, 2017.

[9] S. S. Rao, M. H. Huntley, N. C. Durand, E. K. Stamenova, I. D. Bochkov, J. T. Robinson, A. L. Sanborn, I. Machol, A. D. Omer, E. S. Lander, *et al.*, "A 3D map of the human genome at kilobase resolution reveals principles of chromatin looping," *Cell*, vol. 159, no. 7, pp. 1665–1680, 2014.

[10] F. Chen, G. Li, M. Q. Zhang, and Y. Chen, "Hicdb: a sensitive and robust method for detecting contact domain boundaries," *Nucleic acids research*, vol. 46, no. 21, pp. 11239–11250, 2018.

[11] T. Yang, F. Zhang, G. G. Yardimci, F. Song, R. C. Hardison, W. S. Noble, F. Yue, and Q. Li, "Hicrep: assessing the reproducibility of hi-c data using a stratum-adjusted correlation coefficient," *Genome research*, pp. gr–220640, 2017.

[12] E. P. Consortium *et al.*, "Identification and analysis of functional elements in 1% of the human genome by the encode pilot project," *nature*, vol. 447, no. 7146, p. 799, 2007.

[13] P. H. Sudmant, T. Rausch, E. J. Gardner, R. E. Handsaker, A. Abyzov, J. Huddleston, Y. Zhang, K. Ye, G. Jun, M. H.-Y. Fritz, *et al.*, "An integrated map of structural variation in 2,504 human genomes," *Nature*, vol. 526, no. 7571, pp. 75–81, 2015.

[14] P. H. Sudmant, J. Huddleston, C. R. Catacchio, M. Malig, L. W. Hillier, C. Baker, K. Mohajeri, I. Kondova, R. E. Bontrop, S. Persengiev, *et al.*, "Evolution and diversity of copy number variation in the great ape lineage," *Genome research*, vol. 23, no. 9, pp. 1373–1382, 2013.
